# Subcellular Alterations Induced by Cyanotoxins in Vascular Plants—A Review

**DOI:** 10.3390/plants10050984

**Published:** 2021-05-14

**Authors:** Csaba Máthé, Márta M-Hamvas, Gábor Vasas, Tamás Garda, Csongor Freytag

**Affiliations:** Department of Botany, Faculty of Science and Technology, University of Debrecen, Debrecen Egyetem tér 1, H-4032 Debrecen, Hungary; gtamas0516@gmail.com (T.G.); fcsongor@me.com (C.F.)

**Keywords:** cyanotoxin, microcystin, cylindrospermopsin, plastid, cell wall, vacuole, cytoskeleton, chromatin, cell death

## Abstract

Phytotoxicity of cyanobacterial toxins has been confirmed at the subcellular level with consequences on whole plant physiological parameters and thus growth and productivity. Most of the data are available for two groups of these toxins: microcystins (MCs) and cylindrospermopsins (CYNs). Thus, in this review we present a timely survey of subcellular cyanotoxin effects with the main focus on these two cyanotoxins. We provide comparative insights into how peculiar plant cellular structures are affected. We review structural changes and their physiological consequences induced in the plastid system, peculiar plant cytoskeletal organization and chromatin structure, the plant cell wall, the vacuolar system, and in general, endomembrane structures. The cyanotoxins have characteristic dose-and plant genotype-dependent effects on all these structures. Alterations in chloroplast structure will influence the efficiency of photosynthesis and thus plant productivity. Changing of cell wall composition, disruption of the vacuolar membrane (tonoplast) and cytoskeleton, and alterations of chromatin structure (including DNA strand breaks) can ultimately lead to cell death. Finally, we present an integrated view of subcellular alterations. Knowledge on these changes will certainly contribute to a better understanding of cyanotoxin–plant interactions.

## 1. Introduction

Where water, nutrients, and light occur, the ancient photoautotrophic microorganisms—cyanobacteria—are also expected to emerge in many of the Earth’s habitats [1]. During eutrophication of freshwaters and global warming, many of their representatives are capable of mass reproduction such as blooms, scums, and biofilms, phenomena with several detrimental consequences [2,3]. Cyanobacteria tend to produce special metabolites that help to improve their living conditions and survival [4]. Nowadays, a significant number of low-molecular-weight metabolites with remarkable bioactivity have been identified. The major chemical groups are carbohydrates, polyketides, peptides, terpenes, alkaloids, and phenoloids [4,5]. Many of them are toxic, known for their detrimental effects before they were purified and toxicological and structural characterization was performed [5,6]. Evidence from environmental poisoning events and animal and human illnesses and death have been reported [7].

The cyanotoxins responsible for human and animal intoxications can be classified into families according to their chemical structures [6]. Except for the 10–20 kDa irritant lipopolysaccharides (LPS), these metabolites are low-molecular-weight toxins ranging from 118 Da to ~1000 Da [5].

**Figure 1:**
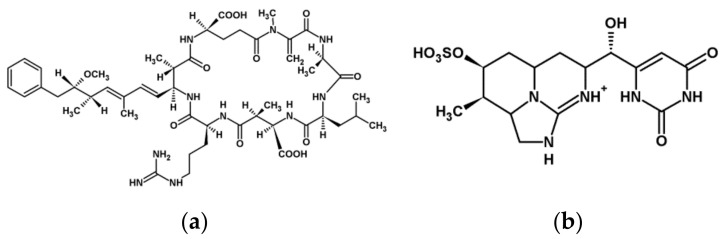
The chemical structure of MC-LR (**a**) and CYN (**b**).

According to their chemical structure and cellular/biochemical targets, the most important cyanotoxins are:

(i) **Microcystins** (**MCs**, Figure 1) are cyclic heptapeptides with inhibitory activity of eukaryotic protein phosphatases PP1 and PP2A and the minor phosphatases PP4 and PP5. They are also known to induce oxidative stress in eukaryotes. At least 279 variants (congeners) of MC have been identified from different cyanobacterial genera such as *Microcystis, Anabaena, Nostoc, Planktothrix,* and *Gleotrichia* [8]. The well-known MC-LR and MC-RR congeners are the most common cyanotoxins responsible for several toxic effects caused by cyanobacteria [5,6,7,9]. Nodularin (NOD) is a pentapeptide with similar effects on MCs [10].
(ii) **Cylindrospermopsins** (**CYNs**, Figure 1) are tricyclic guanidine alkaloids identified among others from *Cylindrospermopsis, Anabaena,* and *Aphanizomenon* species. Their toxicity is characterized by their action on multiple organs, and neurotoxic and genotoxic activity was noted [11]. Their protein synthesis inhibitory activity was published for various organisms [6,7].
(iii) The acetylcholine receptor blocker bicyclic alkaloid **anatoxin-a (ANA)**, the acetylcholine esterase inhibitor phosphorylated cyclic N-hydroxyguanine **anatoxin-a (s)**, and the sodium channel blocker alkaloid **saxitoxins** are neurotoxins produced mainly by filamentous genera [5]. The amino acid type toxin **β-N-methylamino-L-alanine (BMAA)** is also defined as a neurotoxin [12].

While the latter neurotoxic groups affect mainly human and animal tissues and cells, MCs and CYNs, traditionally called hepatotoxins, have a broad spectrum of toxicity since several other organisms (bacteria, fungi, algae, plants) were investigated and toxic consequences were noted [7].

Since the pioneering work of [13], a huge amount of work has demonstrated the detrimental effects of cyanotoxins on aquatic plants and terrestrial crops (see the review of [14] in this topical issue and [15] for examples). For vascular plants, most phytotoxicity data are available for MCs and CYN and thus we concentrate mainly on these two metabolites. Because of their increasing occurrence worldwide, it is timely to review and compare their effects in higher plants at the subcellular level. As we will see, structural and thus physiological alterations induced at this level have crucial consequences on plant growth and productivity. We concentrate on cyanotoxin-induced changes in the organization of plant-specific structures to highlight their different effects on animal/human cells. These peculiar structures are [16]:

(i) the presence of plastids. As mitochondria, their evolutionary origin is explained by the endosymbiotic theory [17]. The occurrence of these semiautonomous organelles re-shapes compartment interactions in the plant cell. In addition, chloroplasts have a central role in plant life since they are the sites of primary production—oxygenic photosynthesis in plants. (ii) Particularities of cytoskeletal organization. Concerning microtubules (MTs), these are the cortical microtubules (CMT), preprophase band (PPB), the acentriolar spindle, and the phragmoplast. The latter mediates Golgi-vesicle delivery of cell wall matrix materials to the division site during mitosis. These vesicles will fuse, forming new plasma membrane surfaces and the cell plate-the rudiment of the future cell wall. This is a complex and highly regulated process that re-shapes vesicle traffic inside the plant cell. Microfilaments have also several particular features and we underline here that organelle dynamics is mostly related to them in plant cells. As cellulose microfibrils are deposited in the cell plate, (iii) the special plant cell wall will be formed, where intercellular connections and macromolecule delivery are achieved by (iv) plasma membrane bridges called the plasmodesmata. (v) Plant cells contain vacuoles with a membrane of particular composition—the tonoplast. An important step in vacuole biogenesis is the formation of multivesicular bodies (MVB), also called prevacuolar compartments (PVC). Both the ER and Golgi-apparatus contribute to the formation of PVC that corresponds to the late endosome of animal cells. Both endocytotic and secretory pathways contribute to their formation.

Cyanotoxin-induced structural alterations will be presented in the structure–function relationship context. Although we will treat toxin effects on the above peculiar plant structures in separate subsections, it should be noted they function in close relationship to each other to integrate different compartments in a eukaryotic cell. Thus, if a metabolite affects a eukaryotic cell compartment, this will have consequences on whole cell functioning. As we will see, many cyanotoxin-induced alterations lead ultimately to cell death. Therefore, we will dedicate a separate subsection to programmed cell death and senescence. Finally, concluding remarks will offer a general view on subcellular alterations and show their consequences at the whole plant level.

There is a huge amount of available data on the effects of cyanotoxins in plants. The vast majority of papers deal with the oxidative stress responses of plants to cyanotoxins (see [15] and references therein for characteristic examples). However, we discuss here only works directly related to their interference in the cell structure–function context. We will concentrate on the effects of cyanotoxins on vascular plants, and thus effects on eukaryotic algae will not be discussed here. Knowledge on the subcellular effects of cyanotoxin on plants is important for a better understanding of the phytotoxicity of these metabolites. Research in this field will certainly contribute to selection of the proper methods for combating their adverse effects on crops.

## 2. Alterations Induced by Cyanotoxins on Peculiar Plant Structures

The “omics” approach is important in estimating cellular alterations induced by cyanotoxins. In this context, the work of Freitas et al. [18] should be cited. Their proteomics studies performed on the vegetable crop lettuce (*Lactuca sativa*) (treated for five days with an environmentally relevant sub-micromolar range of toxin concentrations) revealed many important changes induced by CYN and an MC-LR/CYN mixture. The majority of changes were observed in the MC + CYN combination experiments. Several important changes in protein abundance showed a characteristic “dualistic response”: lower toxin concentrations increased, while higher concentrations decreased or completely abolished their occurrence. In the context of this paper, the most important changes were: (i) in the abundance of proteins related to photosynthetic activity as an important chloroplast function; (ii) chaperone-related plastid and ER proteins involved in protein folding and stress resistance. This raised the possibility that the toxin combination induces structural alterations in these compartments; (iii) proteins involved in cytoskeletal assembly and biosynthesis of cell wall macromolecules; (iv) proteases, raising the possibility of cell death induction. All these changes in the protein pool of cells of treated plants raised the possibility that the toxins and their combination induce alterations in the structure and function of plant cells.

Table 1 summarizes the current knowledge on the subcellular effects of MCs and CYN, the most important cyanotoxins in the context of their interaction with vascular plants.

### 2.1. The Plastid System

One of the pioneering works concerning the effects of MCs on chloroplast functioning was that of Abe et al. [44]. They found rapid, but reversible inhibition of photosynthesis at 10 µM MC-LR and a stable inhibition at 100 µM MC-LR in bean (*Phaseolus vulgaris*) leaves. Under long-term toxin exposure, leaf necrosis was observed.

To date, there are no convincing data on the possible detrimental effects of MCs on the structure of *already existing* thylakoid membranes or any other internal membrane types of plastid. These alterations are expected due to (i) the abundance of data on the inhibitory effects of MCs and other cyanotoxins on photosynthetic oxygen production and photochemical activities, processes that occur at the level of thylakoids and (ii) the involvement of the PP2A–target of MCs in oxidative stress responses in chloroplasts [44,45,46]. However, there is indirect evidence for the interference of MCs with thylakoid *biogenesis*. In mature chloroplasts, where thylakoid membranes do not form a continuum with the membrane envelope, thylakoids are maintained by vesicular transport, where vesicles derive from the inner membrane envelope. This is believed to resemble the mechanisms of eukaryotic vesicular traffic even though plastids are of prokaryotic origin. Besides that, it was proven that this mechanism is present only in embryophytes and thus it is a relatively late evolutionary achievement [47]. The authors proposed that a protein phosphatase–calmodulin complex is involved in vesicle fusion, as for yeast homotypic membrane fusions. Interestingly, MC-LR inhibited this fusion process and this was attributed to its potent inhibitory effects of both PP1 and PP2A [21,47]. It should be noted however, that this effect was observed at a very high concentration of MC-LR (100 µM).

**Figure 2:**
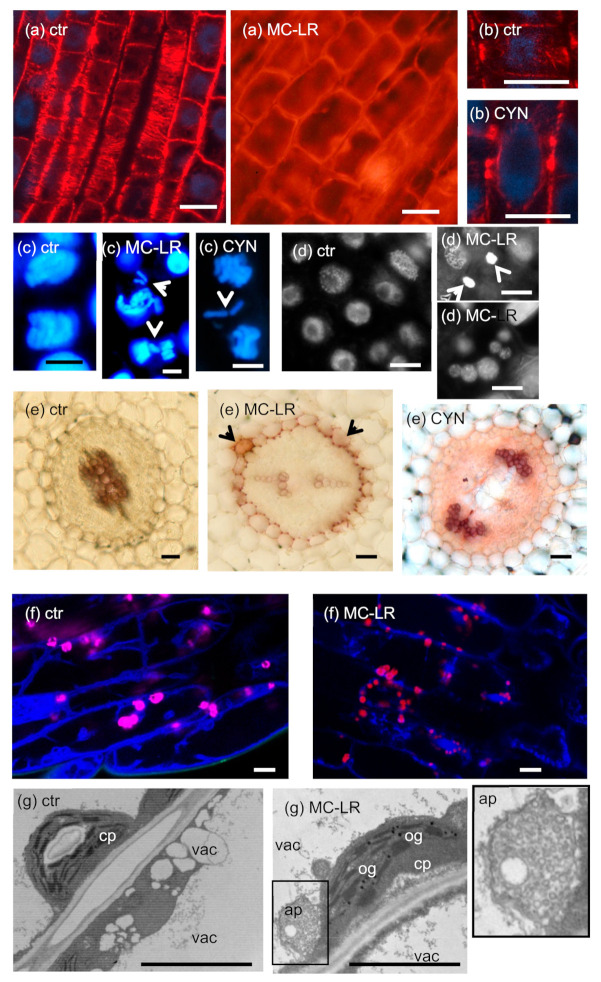
Characteristic subcellular alterations induced by MC-LR and CYN. Panels (**a**–**c**) etc. refer to distinct subcellular features; toxin treatments are presented alongside with controls (ctr). (**a**) *Phragmites australis* roots, control cells present normal cortical microtubules (CMTs), while two day-treatments with 20 µM MC-LR induce depolymerization of CMTs and radial swelling of cells. (**b**) 10 µM CYN induces the formation of double preprophase bands (PPBs) in *P. australis* roots. (**c**) Long-term treatments with high (≥5 µM) concentrations of both MC-LR and CYN induce the formation of lagging chromosomes during cytokinesis and in general, mis-segregation of sister chromatids (arrowheads) in roots of *Vicia faba*. (**d**) 40 µM MC-LR induces chromatin condensation in roots of *P. australis* (upper image), and 10 µM MC-LR induces the formation of numerous nuclear fragments in roots of *V. faba*. Control nuclei are from roots of *P. australis*. (**e**) Phloroglucinol-HCl labels lignin purple. Only walls of xylem cells are labeled in control *Sinapis alba* roots, while 5 µM MC-LR induces uniform labeling of endodermal cell walls (arrowheads), and 10 µM CYN induces cell wall lignification in the whole stele. The inhibition of xylem differentiation by MC-LR and cell swelling in pith tissue by CYN are noteworthy. (**f**) Vacuolar systems of young *Arabidopsis* hypocotyl cells shown by tonoplast labeling with the fluorescent dye CACAIN and chloroplast autofluorescence (pink pseudo-coloring). Controls show vacuoles of different sizes, while 4-h treatment with 5 µM MC-LR induces strong fragmentation of vacuoles. (**g**) TEM images of young *Arabidopsis* hypocotyl cells. 24-h treatment with 2 µM MC-LR induces the formation of osmiophilic granules (og) in chloroplasts (cp) and the formation of autophagosome-like structures (ap). This latter structure is boxed and shown in detail (image on the right) to show its double-membrane envelope and many membrane vesicles inside. vac-vacuole. Scalebars: 50 µm (**a**,**e**), 10 µm (**b**,**d**,**f**), 5 µm (**c**,**g**). Microscopic images collected by L. Székvölgyi (a/ctr), J. Roszik (**b**), C. Máthé (a/MC-LR, c,d), M. M-Hamvas (**e**), Gy. Vereb (**f**), and K. Bóka (**g**). These micrographs were not published previously, and all authors agreed to their publication here.

As concerning the chloroplast stroma, long-term (14 d) application of 5 nM MC-LR and 30.26 µM ANA alone or in combination induced the accumulation of osmiophilic granules in the chloroplasts of *Vallisneria natans* and this was related to the onset of senescence [19]. Exudates and extracts of MC-LR containing *M. aeruginosa* cultures had similar effects related to the generation of reactive oxygen species (ROS) by the toxin [20]. Purified MC-LR is also able to induce the formation of osmiophilic granules (Figure 2g).

To date, there are no data on the effects of cyanotoxins other than MCs on plastid ultrastructure.

### 2.2. The Organization of Plant Cytoskeleton and Mitotic Chromatin

In most cases, cytoskeletal alterations can be directly related to the specific biochemical effects of cyanotoxins (mainly MCs and CYN in the plant context): inhibition of PP1/PP2A by MCs and inhibition of protein synthesis by CYNs. Meanwhile, changes in the expression of proteins building up the cytoskeleton were reported. For example, 0.1 µM MC-LR and crude, MC-LR-containing cyanobacterial extracts induced significant increases in the amount of G-actin in tomato plants [48]. We summarize below cyanotoxin effects on specific cytoskeletal structures.

(i) **CMTs** are important in determining plant cell shape and direction of cell growth by regulating the orientation of cellulose microfibrils in the cell wall [49]. The effects of MCs are largely species- and organ-dependent. In primary roots of the aquatic macrophyte *Phragmites australis* (common reed), both low and high MC-LR concentrations induced CMT disruption (Figure 2a), while in *Ceratophyllum demersum* (coontail) shoots, CMT was not depolymerized, but reoriented. Both changes were attributed to changes in the phosphorylation state and thus functioning of microtubule-associated proteins (MAPs) [23,50]. Differences in the effects of MCs in different species/organs may be related to different tubulin isoforms and MAPs affected. Both types of alterations induced a shift from longitudinal to radial expansion of cells, inducing deformed shapes of whole organs [23,50].
  For CYN, a study on *P. australis* roots showed reorientation and decreased density of CMTs that led to the cessation of cell growth [33]. Protein synthesis inhibition was likely to play a role in this, since the amount of MAPs that regulate MT stability decreased [33]. On the other hand, it is surprising that Western-blots of protein extracts from *Phragmites* roots proved that CYN increased the amount of β-tubulin protein in roots [33]. This was not the case for G-actin; CYN decreased its amount in lettuce leaves [18].
(ii) **PPB** is important in the determination of the orientation of the mitotic spindle and thus regulation of the division plane position [49]. No effects of MCs on PPB MT organization in dicot plants have been detected to date. However, Pappas et al. [28] detected disruptions in PPB assembly in rice root cells under short-term exposure to a very high concentration of MC-LR. This alteration resembled preprophase anomalies in the *fass* mutants of *Arabidopsis*, defective in the B” subunit of PP2A [51].
  CYN induced the formation of double and split PPBs in roots of the dicot *Vicia faba* (broad bean) and the monocot *P. australis* [27,33] (Figure 2b). For broad bean, this led to misorientation of mitotic divisions in root tip meristems [27]. Such PPB anomalies were observed in the presence of the well-known protein synthesis inhibitor cycloheximide [52].
(iii) As for all eukaryotic cells, the acentriolar plant **mitotic spindle** is normally bipolar. MC-LR induces spindle disruptions in a wide variety of species, both dicots and monocots, with the formation of tripolar spindles as a common feature [22,23,25,29]. A wide variety of spindle anomalies (multi-and monopolar, C-and S-shaped, asymmetric, and completely disrupted spindles) were observed in root tip meristems of *Vicia faba* [25]. All these alterations were attributed to an altered phosphorylation state of proteins (e.g., MAPs) that regulate spindle assembly and lead to abnormal sister chromatid segregation during mitosis.
  Spindle disruptions were observed for CYN as well, and in *P. australis* root tip meristematic cells, they were accompanied by lagging chromosomes [29,33]. Although it is likely that protein synthesis inhibition played a role, it should be noted that CYN inhibits protein phosphatase activities in vivo to some extent in *Sinapis alba* (white mustard) seedlings as confirmed by Máthé et al. [29] Meanwhile, CYN has no such effect in vitro [29].
(iv) As we have seen in the introductory section, the **phragmoplast** is crucial for building up the new cell wall during cytokinesis. Both MC-LR (for *P. australis* and *V. faba*) and CYN (for *P. australis*) induce phragmoplast disruptions in dividing root cells [22,23,33]. There is no evidence for abnormal cell plate formation after these disruptions.
(v) **microfilaments (MFs) of non-dividing cells**. A very high (45 µM) concentration of MC-LR induced reorientation and then depolymerization of MFs in root protodermal and differentiated cells of *Oryza sativa* (rice) in very short-term (maximum 1 h) treatments [28]. There are no data on cyanotoxin-induced MF alterations in the mitotic apparatus of plant cells.

The work performed by Pappas et al. [26] showed that crude cyanobacterial extracts induced alterations in MT and MF organization even when they did not contain MCs. The authors proposed the presence of other bioactive peptides than MCs in these extracts capable of inducing such changes [28].

Cyanotoxin-induced changes in the chromatin of non-dividing cells will be shown in the “Plant cell death” Section 2.5; here, we present only mitotic chromatin. As related to changes in the mitotic microtubule apparatus (see above), studies on both dicots and monocots show mis-segregation of sister chromatids following MC treatments (see Table 1). The mechanisms known to date are not only spindle and phragmoplast disruptions, but hyperphosphorylation of histone H3 as well. Phosphorylation of H3 at specific Ser and Thr residues is known to regulate the condensation of chromatin and prevents premature metaphase/anaphase transition [53,54]. MC-LR induced hypercondensation of chromosomes; this was related to the inhibition of PP1 and/or PP2A [22,30]. According to observations on maize meiocytes, PP1 seems to be the main phosphatase responsible for pH3 dephosphorylation [55] and thus the primary target of MCs is possibly this type of phosphatase in this context. As a consequence, lagging chromosomes were detected in root tip meristems of *V. faba* that formed micronuclei at the completion of mitosis [22] (Figure 2c). As we will see at Section 2.5, micronuclei/nuclear fragmentation can be formed during cyanotoxin-induced DNA strand breaks that lead to cell death as well. Interestingly, histone H3 hyperphosphorylation was observed in shoot tip meristems of *Ceratophyllum submersum* naturally co-existing with an MC-containing cyanobacterial bloom in a freshwater pond [39]. Related to this, induction of chromosome aberrations and the formation of micronuclei were reported in the *Allium cepa* (onion) test. Purified MC-LR had significant effects, but these alterations were more pronounced when onion plantlets were treated with MC-LR containing freshwater samples. Extracts of a cyanobacterial culture containing both MC-LR and MC-RR had similar effects [31,32].

Given the effects of MCs on mitotic cytoskeleton and chromatin organization, it is likely that this family of cyanotoxin affects mitotic activity in general. For root tip meristematic cells of *Vicia faba*, a characteristic dual effect was observed. After synchronization of root cells in G1/S and washout of hydroxylurea, 1 µM MC-LR delayed the onset of metaphase–anaphase transition, while 10 µM MC-LR accelerated mitosis within 24 h. In long-term treatments of *S. alba* and *V. faba* seedlings, MC-LR stimulated mitosis at low concentrations and for *S. alba*, inhibited cell division at higher concentrations in non-synchronized root tip meristematic cells [22,29]. These changes were correlated with both cytoskeletal alterations and histone H3 hyperphosphorylation [22,30]. For *P. australis*, no such dualistic effect was observed, since only inhibition of mitosis was detected [23]. For rice roots, purified MC-LR did not block cells in early mitosis, but this happened in the presence of a cyanobacterial extract containing multiple MC congeners [28]. Crude extracts of *Microcystis aeruginosa* with high MC levels stimulated mitosis in *A. cepa* roots and induced the sporadic occurrence of micronuclei. Interestingly, this happened in treatments with cyanobacterial extracts containing members of the aeruginosin family of peptides as well [34].

As shown above, CYN is also able to induce the formation of lagging chromosomes in relation to disruptions in the mitotic cytoskeleton. This was confirmed for both *P. australis* [33] and *V. faba* (Figure 2c). The molecular mechanisms related to this remain to be elucidated. Interestingly, CYN did not inhibit mitosis, but blocked it at early stages in roots of *P. australis*. Thus, even though some cells could pass through the metaphase/anaphase transition, those cells frequently exhibited mis-segregation of sister chromatids [33]. In the case of *V. faba*, the effects of CYN on mitosis depended largely on the time of CYN exposure to seedlings. Under long-term (six days) exposure, the toxin had a dual effect on seedlings cultivated in continuous light: 0.024–0.24 µM CYN stimulated, while 6–48 µM CYN inhibited mitosis. Lagging chromosomes were observed as for *P. australis* and in addition, chromosome breaks frequently appeared in the presence of CYN [27]. This indicates that the genotoxic effects of CYN described for mammalian cells [56] might be universal for eukaryotes. In hydroxylurea-synchronized root cells, the toxin delayed the onset of mitosis in correlation with the delay of *de novo* protein synthesis [27].

At the end of this subchapter, we must point out the tissue-level disorders induced by both MC-LR and CYN regarding local induction of cell dedifferentiation. Both cyanotoxins induce proliferation of cells in the root cortical parenchyma to form a callus-like tissue in *S. alba* and *P. australis*. These cells will show isotropic instead of longitudinal growth [24,33,35]. For *P. australis*, this is a mechanism that obturates aerenchyma tissue of submerged organs to impede their aeration and slow down root metabolic processes. This symptom seems to be a defense response not directly related to the biochemical targets of cyanotoxins. However, it is likely to be related to local disorganization of cytoskeleton and perturbations of cell cycle regulation.

### 2.3. Plant Cell Wall and Plasmodesmata

According to our best knowledge, there are no convincing data on the effects of MCs and CYNs on the ultrastructure of primary plant cell wall.

A combination of MC-LR + CYN induced a decrease in the expression of lettuce leaf xyloglucan endotransglucosylase (XET), an enzyme involved in remodeling of the primary cell wall [18]. The XET enzyme family contributes to remodeling of hemicellulose-cellulose connections in the young cell wall and thus to flexibility and expansion of walls. This allows expansion of the whole cell during elongation [57]. The above inhibitory effects of cyanotoxins might contribute to the general inhibition of plant cell growth observed in many studies cited in the present work as well. As concerning other changes in cell wall composition, the most important alterations detected are:

(i) lignifications. This was observed in the endodermis and stele of MC-LR treated *S. alba* and cortical parenchyma of *P. australis* primary roots. CYN induced partial lignifications in *S. alba* endodermis [29,35] (Figure 2e). Strong lignifications were observed in lateral buds of tissue culture-regenerated *P. australis* stems treated with MC-LR [35]. What is the physiological consequence of such alterations? Lignifications usually occur during secondary wall thickening in dicots and accelerated during thickening in monocots (gramineous plants have a Type II primary cell wall that contains phenylpropanoids *ab ovo*) [57]. The formation of phenylpropanoid polymers is frequently accompanied by obturation of plasmodesmata that finally leads to cell death [57]. These alterations induced by cyanotoxins seem to be non-specific stress reactions (“side-effects”) that occur under long-term exposure to high concentrations (5 µM and above for MC-LR; 24 µM and above for CYN) of cyanotoxins [24,29,35]. The lignification of endodermal cell walls blocks the symplastic pathway of nutrient uptake by roots. This can be considered as a defense response to inhibit the transport of toxins towards shoots.
(ii) callose formation and deposition in tracheary elements. MC-LR induce sporadic deposition of cell wall callose materials in the vascular tissue of *P. australis* roots. Callose obturates tracheary elements that impede the transport of water and minerals (Máthé et al., unpublished data). This is a non-specific stress response. Vascular blockages are observed during organic acid contamination of roots at reed die-back sites as well [58].

### 2.4. The Plant Vacuolar System and Other Endomembranes

The ER and Golgi systems play crucial roles in vacuole biogenesis [59]. Extracts of *Microcystis flos-aquae* TAU-MAC 1510 containing multiple MCs induced aggregations of ER-and Golgi membranes, altering the even distribution of Golgi stacks in rice root cells [28]. Thus, it is likely that MCs affect the vacuolar system as well. Indeed, MC-LR induced fragmentation of the large vacuoles (Figure 2f) in differentiated young hypocotyl cells of *Arabidopsis thaliana* [36]. Since this phenomenon was observed at short-term (4 h) toxin exposures, the above effect is likely to originate from the effects of the toxin on PP2A/PP1. It is worth mentioning that knockout of a PP2A isoform in yeast inhibits vacuole fragmentation and blocks subsequent lysosome formation [60]. Thus, phosphatase inhibition may influence organization of the plant vacuolar system by a pathway distinct to yeast. In parallel with vacuole fragmentation, many chloroplasts appeared to be engulfed in tonoplast-coated small vesicles, presumably functioning as lytic vacuoles “designed” to degrade plastids. This phenomenon occurs in control cells as well, but its frequency increases in the presence of MC-LR [36]. We will return to this issue at the next (Cell death) section.

Plant vacuoles are also thought to participate in the detoxification of MCs. Specific GST enzymes conjugate this toxin to glutathione in chloroplasts and the cytosol, then presumably accumulate in vacuoles [61].

### 2.5. Plant Cell Death

MC and CYN induced subcellular alterations shown above–especially at exposures to high toxin concentrations and/or long-term treatments—anticipate their cell-death-inducing effects. These are cytoskeletal, chromatin, and vacuolar disruptions as well as changes in the composition of cell walls. Indeed, there is a significant number of studies showing necrotic and/or programmed cell death (PCD) occurring in the presence of these cyanotoxins in vascular plants (Table 1).

The first data on cyanotoxin-induced cell-death were the description of browned organs of **MC-**treated *S. alba*, *Phaseolus vulgaris,* and *Brassica napus* seedlings [62,63,64], and of necrosis of leaves of *Phaseolus vulgaris* as a result of dipping leaves into MC-LR solution and on potato shoot cultures growing on MC-LR-containing medium, respectively [44,64]. MC-LR induced necrosis was showed by histological investigations of *S. alba* seedlings’ cotyledons, and cell death was supported by elevated nuclease activity [37]. Chlorosis/necrosis of leaves or/and browning/necrosis of roots as frequent characteristic morphological features of MC treatments have been described for many plant species [24,61,65,66,67].

MCs can cause both apoptosis and necrosis in in vitro animal (for example, lymphocyte) cell cultures in a dose- and time-dependent manner [68]. Cell death processes of plants have several particularities due to their distinctive structures. For example, chloroplasts are a source of ROS as well because of their SOD isoforms and the presence of proteases, nucleases of prokaryotic origin, and thus contribute to the signal-transduction pathways of cell death processes. The name “Apoptotic-like programmed cell death” (AL-PCD) underlines the differences between apoptosis of animal cells and PCD in plants [69].

Yin et al. [42] showed that in MC-RR (50 µM)-treated tobacco BY-2 suspension cells in parallel with the increase in **ROS accumulation decreased cell viability** to approximately 80% was observed after treatment for 144 h. In extracts of cells, elevated POD and GPX activities and changes in SOD and CAT activities were measured. The structural changes in MC-RR treated tobacco BY-2 suspension cells were **perinuclear chromatin margination, condensation of nuclear chromatin, and detachment of the plasma membrane from the cell wall** after 6 d exposure of 50 µM MC-RR. This was the first evidence of MC-induced plant cell apoptosis/PCD. MC-RR-induced PCD in a dose- and time-dependent manner as shown by flow cytometry analysis of propidium iodide (PI) staining (≥1 µM MC-RR/LR/8 d) [43]. MCs are specific inhibitors of protein-phosphatases type 1 and 2A, and in direct or/and indirect connection with this, induce oxidative stress processes in plants [70]. There are numerous data of MC-induced oxidative stress in different crops and aquatic plants, showing alterations of enzymatic and non-enzymatic ROS scavenger systems with plant species-dependent efficiencies (see [24,71]). Huang et al. [72] used a higher dose of MC-RR (60 µM for 5 d) in tobacco with the BY-2 cell system to investigate the role of ROS in MC-induced plant cell apoptosis. MC-RR treatments caused rapid apoptosis: abnormal **elongation of tobacco cells, chromatin condensation and margination, fragmentation of nucleus and formation of apoptotic-like bodies**. There was a significant and rapid increase in ROS levels before the loss of mitochondrial membrane potential (DWm). Application of the antioxidant ascorbic acid and of the specific mitochondrial permeability transition pores (PTP) inhibitor (cyclosporin A) blocked the loss of DWm, as well as cell apoptosis and proved “that the mechanism of MC-RR-induced apoptosis signalling pathways in tobacco BY-2 cells involves not only the excess generation of ROS and oxidative stress, but also the opening of PTP, inducing loss of mitochondrial membrane potential” [72], which is known to be the onset of cell apoptosis [69].

Studies performed in the subsequent years on a wide variety of **crops and hydrophytes** treated with **MC-LR** showed the features of both **apoptosis/AL-PCD** -chromatin marginalization and condensation, nuclear shrinking, blebbing, fragmentation-, and **necrosis** -CMT disruption leading to radial expansion of cells, formation of callus-like tissues, DNA fragmentation (smearing), brownish cells in which autofluorescence fades away and disappearance of nucleus, swollen chloroplasts and mitochondria with destroyed inner membrane structures and outer membranes, and vacuolization of meristematic cells [24,29,35,40,73,74] (Figure 2a,d). **The dose dependent effects of MCs in plant cell death** induction, that is, MCs induce AL-PCD at lower concentrations, while they induce necrosis at higher concentrations or applied for a longer period, are detectable in plants as well [24,73]. It is important to note that the transition from apoptosis to necrosis is also characteristic [40]. As we showed in Section 2.4, vacuolar fragmentation and capturing of chloroplasts in small tonoplast-coated vesicles were detected in MC-LR treated (1–5 µM) *Arabidopsis* hypocotyls (Figure 2f). Prolongation of treatments (72 h) increased the number of multimembrane vesicles as well as autophagosome-like structures, which were incorporated into the vacuoles [36]. Formation of autophagosomes seems to be typical for the initial stages of several cell death processes in plants [75]. Jámbrik et al. [40] reported that MC-LR-induced PCD is associated with changes in single-strand-preferring **SSP nuclease** activities in *Phragmites australis* cells, in connection with the condition of nucleus. These changes in nuclease activities were correlated with DNA repair and degradation processes.

Water-bloom samples with MC-LR contents significantly elevated the portion of root tip meristematic cells of *Allium cepa* having symptoms of cell death detected on the basis of increased cellular volume, peripherally positioned nucleus and/or vacuolated cytoplasm [31].

Toxic water-bloom induced cell death was proved in the real environment as well. *Ceratophyllum submersum* collected from MC-dominated harmful algal blooms (HABs) showed decreases in anthocyanin and carotenoid contents and POD activity that indicated reduced ROS scavenger activities. As a result, histological/cytological alterations, radial swelling and abnormal development of shoots, fragmentation of nuclei and accumulation of phenolics in the nucleus, necrotic cells with abnormal cell wall thickening, and with absence of DNA were detected. The further indications of HAB-induced cell death and stress reactions were the increases in nuclease and protease activities showing the dominance of degradative processes [39].

The early aerenchyma formation and lignification of cortical parenchyma, endodermis, and pericycle cells could be signs of accelerated differentiation/senescence, which were detected in the MC-LR treated reed and mustard, and in mustard treated with high CYN concentrations [24,29,35] (Figure 2e).

All these alterations induced by MCs are difficult to be correlated directly with their protein phosphatase activity. It seems that PCD/necrosis is induced by inducing non-specific, oxidative stress-related effects by of these cyanotoxins.

**CYN** induced oxidative stress and alterations in enzyme activities (ROS scavengers, nucleases and proteases) in *S. alba, Oryza sativa* and *Lemnaceae* species [38,76,77,78]. A comparative study of histological effects of wide concentration ranges of CYN vs. MC-LR in *S. alba* seedlings showed chlorosis without necrosis and cell expansion in the root’s pith instead of cortex [29]. Prolonged exposure (6–9 days) to crude cyanobacterial extracts with 6 µM CYN induced decreases in water contents, cell expansion, green color, and browning of *Oryza sativa* leaves [78]. Histological and cytological investigations of 12–24 µM CYN-treated reed (*P. australis*) roots revealed the formation of callus-like structure containing radially swollen cortical and rhizodermal cells and the necrosis with a lack of autofluorescence in these tissues. However, the early aerenchyma differentiation, which was characteristic in MC-LR treated reeds’ roots, was not detected for CYN [33,35]. Necrotic cells formed a contiguous ring in the rhizodermis and inner and outer cortex of *Vicia* roots treated with 48 µM CYN for 6 d. Time-and dose-dependent blebbing, fragmentation, degradation of nuclei, PCD related micronucleus formation (*S. alba, V. faba*), and chromosome breaks (*V. faba*) in meristematic cells were detected [24,27]. CYN induced an increase in the ratio of TUNEL-positive cells with chromatin fragmentation in reed. This was correlated with alterations in nuclease and protease isoenzyme (the two main families of hydrolases with triggering and regulatory functions in PCD) patterns and activities in mustard seedlings. These findings added further evidence of CYN-induced PCD in vascular plants [41]. Future results may contribute to the elucidation of the mechanisms of CYN-induced plant cell death processes, for example, CYN-induced cell death could be mediated not only through ROS generation, but by inhibition of pyrimidine nucleotide synthesis as was shown in different animal cell systems [79].

## 3. Conclusions

**Figure 3:**
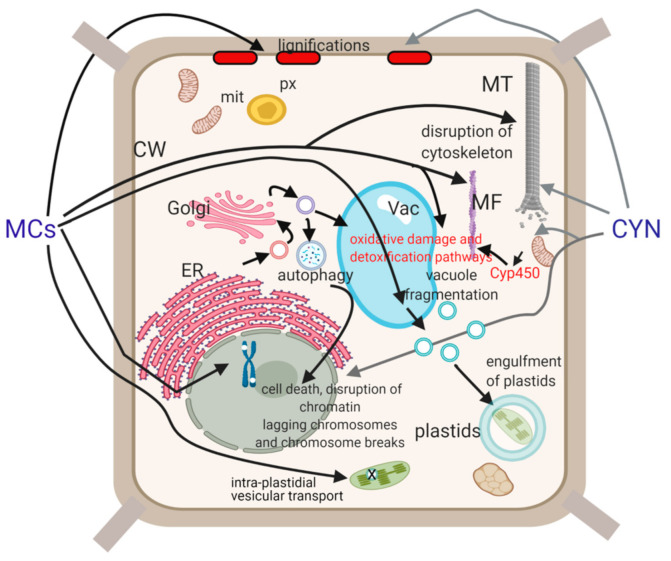
A summary of subcellular effects for microcystins (MCs) and cylindrospermopsin (CYN). Physiological alterations such as the detailed mechanisms of oxidative damage or inhibition of chloroplast photosynthetic activity are not shown here. CW—cell wall, Cyp450—cytochrome monooxygenase P450, MF—microfilament, mit—mitochondria, MT—microtubule, px—peroxisome, vac—vacuole. Image created with BioRender.com.

The subcellular effects of MC-LR and CYN are summarized on Table 1 and Figure 3. In the plant cell death section, diverse subcellular alterations induced by cyanotoxins can ultimately lead to PCD or “non-specific” cell death (necrosis). However, cell death occurs mainly at very high, sometimes environmentally non-relevant toxin concentrations, while low concentrations frequently induce diverse stimulatory effects. This is particularly true for MCs and points to the importance of real cyanotoxin concentrations when one considers their cytotoxic effects. For example, low MC concentrations stimulate mitosis, while high concentrations have a strong inhibitory effect. In addition, these dualistic effects are important in the context of the biochemical targets of cyanotoxins. Their specific effects (e.g., PP2A/PP1 inhibition by MCs) are exerted mainly at low concentrations/short exposure times, while high concentrations/long exposure times may cause non-specific (general stress response) effects. However, many subcellular effects of MCs summarized in this review are likely to be related to phosphatase inhibition.

The detrimental effects of both MCs and CYNs on plant cytoskeleton have a distinct importance, since this structure is crucial for the integrated functioning of a eukaryotic cell. Now we know many effects of MCs on MTs and MFs and those of CYNs on MTs in plants. These studies underline that MT and MF disruptions will alter cell division and cell shape, which will ultimately lead to distorted organs and inhibition of elongation growth. One of the most interesting changes are the misorientation of mitotic planes by disruption of normal PPB organization by CYN. Alteration of CMT organization will alter cell shape and elongation growth in MC and CYN treatments. Proper growth and development of a given organ is of course a complicated process dependent on many subcellular/physiological processes. For example, a recent study has shown that MC-LR changes the distribution of PINs/auxin efflux carriers, hence auxins in roots of *Arabidopsis*. As a consequence, elongation of primary roots is inhibited and lateral root development altered [80]. Perhaps changes in local auxin amounts within roots are correlated with misorganization of MTs, but the understanding of this relationship needs further research in general and in the cyanotoxin context in particular.

It is also clear that given the impact of cyanotoxins on aquatic ecosystems and their adverse effects on terrestrial crops (via irrigation with toxin-contaminated water), there is tremendous work to perform in the future for a better understanding of their cellular effects. Here are some directions of study that we consider to be important:

(i) Endomembrane systems such as the ER are as important as the cytoskeleton for the integrated functioning of the plant cell. There is still a lack of knowledge on the relevant effects of cyanotoxins. For example, do cyanotoxins induce ER stress as related to plant cell death?
(ii) Although CYN is considered to be a protein synthesis inhibitory toxin in eukaryotes, we still do not know much on its particular molecular targets. Studies on plant cells might be essential in this issue.
(iii) Most knowledge on the effects of cyanotoxins on plant cells involves MCs and CYNs. What about the other cyanotoxins? Non-MC peptides such as aeruginosins, microginins, etc., are of particular interest because many of them are protein phosphatase inhibitors, proteases or protease inhibitors and as such, they are likely to induce subcellular alterations.

## Figures and Tables

**Table 1:** Comparison of the effects of microcystins (MCs) and cylindrospermopsin (CYN) on plants at the subcellular level.

Cell Compartment/Phenomenon	Effects of MCs Including MC Type and Concentration	Effects of CYNs Including CYN Concentration	Mechanisms
**Plastids**	5 nM MC-LR ^a^ + 30.26 µM ANA/14 d MC-LR containing *Microcystis aeruginosa* cultures: accumulation of osmiophilic granules in chloroplasts of *Vallisneria natans* [19,20]	n.d.	Generation of ROS by MC-LR
	100 µM MC-LR ^a^ isolated chloroplasts of pea, inhibition of vesicle traffic in plastids [21]		PP1/PP2A inhibition
**Cytoskeleton**	**MTs**: **CMT**—0.01–40 µM MC-LR ^a^—disruption in *Phragmites australis*, reorientation in *Ceratophyllum demersum* **PPB**—45 µM MC-LR ^a^, short-term exposure—PPB disruption in rice root meristem **spindle**—0.05–40 µM MC-LR ^a^—disruptions, deformations in *Sinapis alba*, *Vicia faba*, *P. australis* **phragmoplast**—0.5–40 µM MC-LR ^a^—disruptions in roots of *P. australis, V. faba* [22,23,24,25,26]	**MTs**: **CMT**—12–96 µM CYN ^a^—reorientation, decrease in their density in *P. australis* **PPB**—2.4–24 µM CYN ^a^—double and split PPBs in roots of *P. australis*, *V. faba* **spindle**—2.4–12 µM CYN ^a^—disruptions, deformations of metaphase and anaphase spindle in roots of *S. alba* and *P. australis* **phragmoplast**—1.2–12 µM CYN ^a^—disruptions in roots of *P. australis* [24,27]	PP1/PP2A inhibition for MCs; protein synthesis inhibition for CYN
	**MFs**—45 µM MC-LR ^a^ and MCs short-term treatment: misorientation of cortical MFs in rice roots [28]	**MFs**: n.d.	PP1/PP2A inhibition?
**Mitotic chromatin, mitotic index**	0.5–40 µM MC-LR ^a^-mis-segregation of sister chromatids including lagging chromosomes in telophase/cytokinesis, micronucleus: *S. alba*, *V. faba*, *P. australis* 0.001–0.002 µM MC-LR ^a^ and MCs ^c^: chromosome aberrations and micronuclei in *Allium cepa* roots 1–10 µM MC-LR ^a^: alterations in the timing of metaphase–anaphase transition MCs ^b^—blocking of cells in early mitosis, rice roots [22,23,25,26,29,30,31,32]	1.2–12 µM CYN ^a^—lagging chromosomes in root tips of *P. australis* 2.4, 6 µM CYN ^a^—blocking of cells in early mitosis in *P. australis* roots 12 µM CYN ^a^—delay of mitosis in synchronized *V. faba* roots 0.24–12 µM CYN ^a^—chromosome breaks in roots of *V. faba* [24,27,33]	disruptions in the mitotic MT cytoskeleton; for MCs, hyperphosphorylation of histone H3 related to PP1 (PP2A) inhibition For CYN, inhibition of protein synthesis?
	MC-LR ^a^—inhibition of mitosis: *S. alba* (≥10 μM)*, P. australis* (≥0.5 μM); stimulation of mitosis at lower concentrations (1 µM): *S. alba, V. faba* MC ^b^: stimulation of mitosis, *A. cepa* roots [23,24,34]	0.024–0.24 µM CYN ^a^—stimulation and 6–48 µM CYN ^a^—inhibition of mitosis in roots of *V. faba* [24,29]	probably related to the direct biochemical targets of cyanotoxins
**Cell wall**	5–40 µM MC-LR—lignification of cell walls in root cortex and stele of *S. alba* and *P. australis* [29,35]	24–48 µM CYN ^a^—lignification of endodermis and pericycle cells of *S. alba* roots [24]	non-specific stress reactions?
**Vacuoles and other endomembranes**	MCs ^b^, aggregations of ER and Golgi membranes in rice root cells 1 µM MC-LR ^a^, short-term exposure: vacuole fragmentation, engulfment of plastids in tonoplast-coated vesicles in *Arabidopsis* hypocotyl cells [8,17]	n.d.	n.d. for ER/Golgi; PP2A/PP1 inhibition for vacuole fragmentation [28,36]
**Cell death**	5–100 µM MC-LR ^a^: cotyledon, leaf and/or root **necrosis**, in *Phaseolus vulgaris, S. alba, Brassica napus, P. australis, Ceratophyllum submersum* −5–10 µM/2–20 d: CMT reorganization caused crown root formation and radial expansion of cells 1 µM: plasmolysis, swollen chloroplasts and mitochondria with destroyed inner membrane structures in *V. natans* [23,29,35,37,38,39,40,41,42]	-root necrosis in *P. australis* (≥24 μM CYN) and *V. faba* (2.4–48 μM CYN, but not in *S. alba* −12–24 µM/2–20 d: swelling of cells and formation of a callus-like tissue *S. alba*, *P. australis* without early formation of aerenchyma [27,29,33]	generation of ROS induced by MCs and CYN; alterations in nuclease (ssDNase and dsDNase) and protease activities [37,38,39,40,41]
	**apoptosis/AL-PCD**: MC-RR ^a^ 60 μM/5 d and ≥1 μM/8 d: TobaccoBY-2 cells, 5 µM/4 d *S. alba* seedlings: perinuclear chromatin margination, condensation of nuclear chromatin, shrinking, blebbing, fragmentation of nucleus, formation of apoptotic-like bodies, the loss of mitochondrial membrane potential (DWm) 1–2 μM MC-LR ^a^/72 h, autophagosome formation in *Arabidopsis* hypocotyl cells [24,36,43,44,45]	In *V. faba* 12–48 µM/3–6 d CYN ^a^ induced nucleus fragmentation, blebbing and chromosomal breaks, and increased the ratio of TUNEL-positive cells in 1.2–48 µM/10 d CYN ^a^ treated *P. australis* and in 0.024–24 µM/4 d CYN ^a^ treated *S. alba* roots, in *P. australis* chromatin fragmentation was detected as well [24,27,41]	
	50 μM MC-LR ^a^/72–144 h reduced cell viability of TobaccoBY-2 cells (Evans blue, PI, staining) Significantly higher cell death index compared with control meristematic *A. cepa* root tip cells [31,42,43]		

Abbreviations: CMT—cortical microtubule; MF—microfilament; MT—microtubule; n.d.- no data; PI—propidium-iodide; PPB—preprophase band; ROS—reactive oxygen species. Type of toxin preparations: ^a^ commercial; ^b^ cyanobacterial extract containing multiple MCs; ^c^ MC containing freshwater samples and cyanobacterial extracts.

## Data Availability

Data for this paper can be found in the references cited.
